# Supplementary material for: A 4D-Printable Photocurable Resin Derived from Waste Cooking Oil with Enhanced Tensile Strength
Source: Molecules. 2024 May 6;29(9):2162. doi: 10.3390/molecules29092162 (PMC11085575; doi:10.3390/molecules29092162)
Supplement: Supplementary file 1 [file molecules-29-02162-s001.zip › Supplementary Materials.pdf]

Supplementary Materials

A 4D-Printable Photocurable Resin Derived from Waste Cooking Oil with Enhanced Tensile Strength

*Yan Liu, Meng-Yu Liu, Xin-Gang Fan, Peng-Yu Wang and Shuo-Ping Chen**

College of Materials Science and Engineering, Guilin University of Technology, Guilin 541004, China

*Corresponding author: Shuo-Ping Chen. E-mail address: chenshuoping_777@163.com. Phone: +86-773-5896672. Fax: +86-773-5896671.

Number of pages: 28

Number of tables: 7

Number of figures: 13

Number of Video: 1

List

S1. Synthesis of acrylate-based WCO

S2. 4D printing of photocurable resin based on WCO

S3. Characterization

Table S1. Fatty acid compositions (wt.%) of five kinds of oil. Three main types of fatty acids: saturated (C n:0), monounsaturated (C n:1), and polyunsaturated with two or three double bonds (C n:2/3).

Table S2. The Lovibond color codes of WCO and E-WCO.

Table S3. The viscosities of E-WCO, pure EWOA, pure EWOA, liquid A2 resin and liquid MA2 resin at room temperature.

Table S4. The mechanical properties of different 4D-printable photocurable resin.

Table S5. The thermogravimetric data of WCO-based 4D-printable resin and other control samples.

Table S6. The glass transition temperatures (T_g) and crosslinking density of WCO-based 4D-printable resins composed of EWOA and different diacrylate molecules.

Table S7. The glass transition temperatures (T_g) and crosslinking density of EWOA/EWOMA-TEGDMA resins with different dosages of TEGDMA.

Figure S1. Photograph of WCO and E-WCO showing the difference in their colors.

Figure S2. The aldehyde contents of WCO and E-WCO.

Figure S3. The absorption spectrum of the WCO-based photocurable resin (A2: blue, MA2: red) and the emission spectrum of the LCD curing light sources (orange).

Figure S4. The influence of exposure time on the 4D-printed product.

Figure S5. The influence of layer thickness on the 4D-printed product.

Figure S6. (a) IR spectra of 4D-printed product and liquid resin of MA2; (b-d) The full XPS (b), C1s (c), and O1s (d) high-resolution XPS spectra of 4D-printed product of MA2 resin.

Figure S7. (a) The stress-strain curves of pure EWOA and WCO-based 4D-printable resins composed of EWOA and different diacrylate molecules; (b) The stress-strain curves of pure EWOA, pure TEGDMA and EWOA-TEGDMA resin with different dosages of TEGDMA; (c) The stress-strain curves of of pure EWOMA, pure TEGDMA and EWOMA-TEGDMA resins with different dosages of TEGDMA; (d-e) The stress-strain curves of A2 (d) and MA (e) resins at different temperatures; (f) The crosslinking density of WCO-based 4D-printable resins composed of EWOA and different diacrylate molecules; (g) The crosslinking density

of EWOA-TEGDMA resins with different dosages of TEGDMA; (h) The crosslinking density of EWOMA-TEGDMA resins with different dosages of TEGDMA.

Figure S8. (a, b) The TG and DTG curves of pure EWOA, pure TEGDMA and EWOA-TEGDMA resins with different dosages of TEGDMA; (c, d) The TG and DTG curves of pure EWOMA, pure TEGDMA and EWOMA-TEGDMA resins with different dosages of TEGDMA.

Figure S9. (a-f) The DMA spectra of EWOA-TEGDMA and EWOMA-TEGDMA resins with different dosages of TEGDMA: (a) A1, (b) A2, (c) A3, (d) MA1, (e) MA2, (f) MA3; (g-i) The DMA spectra of pure EWOA (g), EWOMA (h) and TEGDMA (i).

Figure S10. The dynamic mechanical temperature spectra of EWOA-R resin with different second monomers (both the dosages of EWOA were 100 g in this series)): (a) A2, (b) B2, (c) C2, (d) D2, (e) E2, (f) F2.

Figure S11. (a-f) The DSC spectra of EWOA-TEGDMA and EWOMA-TEGDMA resin with different dosages of TEGDMA: (a) A1, (b) A2, (c) A3, (d) MA1, (e) MA2, (f) MA3; (g-i) The DSC spectra of pure EWOA (g), EWOMA (h) and TEGDMA (i).

Figure S12. The DSC spectra of WCO-based 4D-printable resins composed of EWOA and different diacrylate molecules: (a) A2, (b)B2, (c) C2, (d) D2, (e) E2, (f) F2.

Figure S13. CAD model diagram of 1BA dumbbell-shaped spline according to the China Standard GB/T 1040.2-2006.

Video S1. Video presentation of a shape memory cycle of a 4D-printed radish roots of A2 resin.

S1. Synthesis of acrylate-based WCO

Initially, we epoxidized WCO to obtain E-WCO. To achieve this, we mixed 1240 g of 30% H_2O_2, 320 g of glacial acetic acid, 8 g of sulfuric acid, and 8 g of urea in a lightproof container. This mixture was placed in an oven at 40 °C for 12 hours to generate peracetic acid as the epoxidation reagent. Subsequently, 1000 g of WCO was placed in a glass-stirred reactor and heated to 40 °C. The prepared epoxidation reagent was slowly added to the reactor over 2 hours. The reaction mixture was then heated to 70 °C and stirred for 3 hours. After completing the reaction, the mixture was allowed to stand for 12 hours to stratify. The upper oil layer was collected, washed once with a 5% $NaHCO_3$ solution, and then washed twice with deionized water at 60 °C. Finally, it was vacuum evaporated at 70 °C to yield E-WCO with an epoxy value of 4.37. After the epoxylation reaction, the color of the resulting product significantly improved, changing from a dark brown color (Lovibond color scale: Y = 50.5 and R = 20) to yellow (Y = 10 and R = 1) (See Figure S1 and Table S2 in ESI). Additionally, epoxylation removed over 70% of aldehyde compounds from the oil product, thereby effectively eliminating the unpleasant odor associated with WCO (See Figure S2 in ESI).

Next, E-WCO underwent a ring-opening esterification reaction with unsaturated carboxylic acids (AA or MAA) to produce acrylate-based WCO, namely epoxy waste oil acrylate (EWOA) or epoxy waste oil methacrylate (EWOMA), respectively. In this ring-opening esterification process, 100 g of E-WCO was combined with 0.1 g of HQ in a three-neck flask and stirred to form a homogeneous solution at 90 °C. Simultaneously, 19.7 g of AA (or 23.5 g of MAA) and 1 g of PPh_3 were mixed in a beaker at 60 °C to create another homogeneous solution. The AA (or MAA) solution was gradually added dropwise to the E-WCO solution using a constant pressure funnel. The reaction mixture was then heated at 100 °C for 4 hours with magnetic stirring. The resulting EWOA (or EWOMA) appeared as a brownish-yellow transparent liquid. Both pure EWOA and EWOMA were photocurable under UV light irradiation when an initiator was introduced. However, the high viscosity of pure acrylate WCO (5271 mPa·s for EWOA and 4322 mPa·s for EWOMA) might pose challenges during the 3D printing process, making it susceptible to printing defects due to flow difficulties.

S2. 4D printing of photocurable resin based on WCO

The 4D printing of the WCO-based photocurable resin was conducted using a Photon X LCD 3D printer (Anycubic, Shenzhen, China) as previously described in our work (Yan. Liu, Meng-Yu. Liu, et al., 2022). In order to balance the quality of 4D printed products and printing efficiency (refer to Figures S4 and S5 in ESI), the optimized printing parameters are as follows: a bottom exposure time of 120 seconds, a normal exposure time of 37 seconds, and a layer thickness of 50 μm. Concurrently, the STL files for 4D printing were imported into the LCD 3D printer, commencing the printing process. Upon the completion of printing, a pristine, transparent yellow product featuring a smooth surface and well-defined details was achieved. Subsequently, the 4D printed item underwent a thorough ethanol cleaning procedure to eliminate any residual uncured material from its surface. After air-drying, it was then prepared for immediate utilization or further testing.

S3. Characterization

The characterization of the resulting WCO-based photocurable resins and other related samples was carried out in accordance with the methods detailed in our prior publication.(Y. Liu, M. Liu, et al., 2022) The iodine value of WCO was determined using the China Standard GB/T 5532-2008. The epoxy value of E-WCO was examined following China Standard GB/T 1677-2008. The contents of various fatty acids in the WCO were measured following the China Standard GB 5009.168-2016 (to prepare the methyl esters) using an Agilent 7890-5979 gas chromatography-mass (GC-MS) spectrometer (Agilent, Santa Clara, USA). The Lovibond color of WCO and E-WCO was analyzed by a LABO-HUB WSL-2 Lovibond tintometer (Xinrui, Shanghai, China). The viscosities of pure EWOA, EWOMA and liquid WCO-based resin were checked with an NDJ-8s viscometer (Jitai, Shanghai, China). The UV-VIS absorption spectra of the liquid resin were measured by a UV3100 UV-VIS-NIR spectrophotometer (Shimadzu, Tokyo, Japan). Infrared (IR) spectrum of the resulting products was recorded as KBr pellets in the range of 400–4000 cm^{-1} on a Nicolet 5700 FT-IR spectrometer (Thermofisher, Waltham, USA) with a spectral resolution of 4 cm^{-1}. The X-ray photoelectron spectroscopy (XPS) of the cured resin was carried out with an ESCALAB 250Xi X-ray photoelectron spectrometer (Thermofisher, Waltham, USA) with an Al Kα X-ray as the stimulating source.

The mechanical properties were examined by an AG-20I electronic universal testing machine (Shimadzu, Tokyo, Japan) with a 1 mm/min stretching rate. The test sample was printed as a 1BA dumbbell-shaped spline according to the China Standard GB/T 1040.2-2006, whose CAD model diagram was shown in Figure S13. The results represent the average measurements of four samples having the same composition. The thermogravimetric analysis was carried out with a TG 209 F1 Libra thermal gravimetric analyzer (Netzsch, Selb, Germany) at a heating rate of 10 °C/min in the range of 25−600 °C. The tests of differential scanning calorimetry (DSC) were performed on a DSC 204/2920 differential scanning calorimeter (TA Instruments, New Castle, USA) equipped with a refrigerated cooling system. After equilibrating the samples at −60 °C for 5 min, the first heating trace started from −60 to 100 °C at 10 °C/min. The samples were equilibrated at 100 °C for 1 min and cooled down at 3 °C/min. When the temperature reached −60 °C, the samples were equilibrated for 1 min and heated again to 100 °C at 10 °C/min. The first cooling and second heating traces were recorded to study the glass transition behaviours of resin. The dynamic

thermomechanical analysis (DMA) was carried out using a DMA861e dynamic thermomechanical analyzer (Mettler Toledo, Zurich, Switzerland) in the shear mode under a nitrogen atmosphere in the temperature range of −50 to 150 °C, at a heating rate of 3 ℃/min and a frequency of 1 Hz. The test sample was printed as a rectangular strip measuring 40 mm × 12.5 mm × 2 mm. The crosslinking density (V_e, mol/cm^3) of the sample was calculated from the DMA result using the following equation.

$$V_e = E'/3RT \quad (1)$$

where E' is the energy of storage modulus (G') at T_g+30 °C, R is the gas constant (8.314 J·mol^{-1}·K^{-1}) and T is the absolute temperature of T_g+30 °C. The results of the crosslinking density of EWOA-TEGDMA and EWOMA-TEGDMA resin are shown in Figures S7g and S7h, Tables S9 in ESI.

The shape memory behaviour of the WCO-based 4D-printed product was investigated following a typical shape memory cycling method using a TA Q800 dynamic thermo-mechanical analyzer (TA Instruments, New Castle, USA), where the test sample was printed as a rectangular strip with a size of 25 mm × 5 mm × 0.6 mm. The sample was first stretched by 10% with a constant strain rate of 6 %/min at 25 °C in a nitrogen atmosphere. Then, the temperature was decreased to −60 °C at a cooling rate of 2 °C/min and held isothermally at −60 °C for 3 min. The strain of the temporary shape was measured after removing the external load. In the free recovery step, the temperature was gradually increased to 25 °C at 2 °C/min. The sample was held isothermally at 25 °C for another 45 min to observe the free recovery behaviour. To quantify the shape memory behaviour, the shape fixity ratio

$$R_f = \varepsilon_f/\varepsilon_m \times 100\% \quad (2)$$

and the shape recovery ratio

$$R_r = (\varepsilon_f - \varepsilon_r)/\varepsilon_f \times 100\% \quad (3)$$

were calculated, where ε_m is the maximum strain before unloading, ε_f is the strain right after unloading, and ε_r is the instantaneous strain during recovery.

The shape memory parameters, including the shape memory fixity ratio (R_f) and recovery ratio (R_r) of the WCO-based 4D-printed product, were also tested by the U-shaped bending test method. The 4D-printed product was introduced into a rectangular strip with a dimension of 70 mm × 6 mm× 1 mm, and the testing was carried out as follows: First, the sample was heated at deforming temperature in the water bath for 3 min, so that it becomes soft and flexible. Then, it was bent at a given angle θ_i to form a deformed shape. Secondly, the

deformed shape was quenched to −60 °C (fixing temperature) in a dry ice-ethanol bath and maintained for 3 min, and the fixing angle θ_f was obtained after releasing the bending stress. Finally, the sample was introduced into a water bath at deforming temperature for 3 min. After releasing the residual stress, the recovery angle θ_r was recorded from the recovery of the permanent shape. The values of R_f and R_r were calculated using the following equations:

$$R_f = \theta_f/\theta_i \times 100\%; \quad (4)$$

$$R_r = (\theta_i - \theta_r)/\theta_i \times 100\%. \quad (5)$$

The biodegradability test was evaluated through soil burial tests. Initially, square specimens measuring 10×10×2 mm were printed using a 3D printer and placed in containers filled with garden soil. The specimens were buried 50 mm below the soil surface. Multiple sets of samples (including control samples) were prepared. Subsequently, the containers were placed in a humidity chamber with controlled temperature conditions set at 25°C and 30% relative humidity, maintained for a duration of 45 days. At specific intervals, samples were retrieved, cleaned, and vacuum-dried at 25°C for 24 hours. The weights of the samples were measured before (W_{before}) and after (W_{after}) the biodegradation test, and the biodegradation rate (measured by the weight loss, %) was calculated using the following formula:

$$\text{Weight loss} = (W_{befor} - W_{after})/W_{before} \times 100\%. \quad (6)$$

Table S1. Fatty acid compositions (wt.%) of five kinds of oil. Three main types of fatty acids: saturated (C n:0), monounsaturated (C n:1), and polyunsaturated with two or three double bonds (C n:2/3).

Fatty acid		Supernatant WCO
Caprylic	C 8:0	0.2
Lauric	C 12:0	0.1
Myristic	C 14:0	0.2
Palmitic	C 16:0	19.7
Palmitoleic	C 16:1	0.5
Stearic	C 18:0	6.8
Oleic	C 18:1	69.6
Linoleic	C 18:2	3.0
Linolenic	C 18:3	0.3
Arachidic	C 20:0	0.5
Gadoleic	C 20:1	0.1

Table S2. The Lovibond color codes of WCO and E-WCO.

Sample	Y (Yellow)	R (Red)	B (Blue)	Light field	Darkfield
WCO	50.5	20.3	8.9	0	0
E-WCO	10.2	1.1	0	0	0

Table S3. The viscosities of E-WCO, pure EWOA, pure EWOA, liquid A2 resin and liquid MA2 resin at room temperature.

Sample	Viscosity (mPa·s)
E-WCO	2127
EWOA (control sample)	2426
EWOMA (control sample)	4322
A2	141
MA2	159

Table S4. The mechanical properties of different 4D-printable photocurable resin

Major compositions	Tensile strength (MPa)	Breaking elongation (%)	Reference
Photo-curable acrylate resin (P resin, contained HEA, IBOA, and TMPTA)<br>Thermally curable epoxy resin (T resin, was composed of DGEBA and MTHPA)	59.2	2.50	Chen et al., 2021
Isobornyl acrylate (IBOA)<br>Trimethylolpropane triacrylate (TMPTA)<br>Epoxy acrylate (EA)	26.4	12.6	W. Shan et al., 2020
N-Vinylpyrrolidone (NVP)<br>O-polyethylene glycol diacrylate (PEGDA)<br>Glycidyl methacrylate-grafted polyetherimide (PEI-GMA)	4.3	18.50	Chung et al., 2022
Acryl-morpholine (ACMO)<br>Polyurethane acrylate (PUA)<br>3,4-epoxy cyclohexyl methyl-3,4-epoxy cyclohexyl formate (UV-6110)	28.46	14	J. Shan et al., 2020
Cyclic trimethylolpropane formal acrylate (CTFA)<br>2-phenoxyethyl acrylate (PHEA)	2.8	47.5	Yang et al., 2022
Epoxy waste oil methacrylate (EWOMA)<br>2-phenoxyethyl acrylate (PHEA)<br>Methacrylic acid (MAA)	0.48	230.1	Y. Liu, M. Liu et al., 2022
Epoxy waste oil acrylate (EWOA)<br>triethylene glycol dimethacrylate (TEGDMA)	9.17	15.39	A2 in this work
Epoxy waste oil methacrylate (EWOMA)	10.74	10.52	MA2 in this work

triethylene glycol dimethacrylate (TEGDMA)	

Table S5. The thermogravimetric data of WCO-based 4D-printable resin and other control samples.

Sample	Initial decomposition temperature (℃)	The temperature at which the decomposition rate is maximum (℃)	Peak decomposition rate (%/min)	The final carbon residue (%)
A1	278.92	416.32	10.99	4.32
A2	283.01	420.81	9.59	3.8
A3	273.03	420.13	10.29	2.19
MA1	266.91	416.51	11.31	6.34
MA2	282.31	418.61	12.69	5.44
MA3	241.14	405.14	8.59	5.71
EWOA (control sample)	247.92	383.62	8.825	4.985
EWOMA (control sample)	261.44	408.15	9.46	2.25
TEGDMA (control sample)	252.88	304.89	9.72	2.59

Table S6. The glass transition temperatures (T_g) and crosslinking density of WCO-based 4D-printable resins composed of EWOA and different diacrylate molecules.

Sample	Glass transition temperature (T_g, ℃)	Crosslink density (mol/cm^3)
B2	39.25	1.7×10^{-3}
C2	33.16	1.63×10^{-3}
A2	21.65	2.41×10^{-3}
D2	27.33	1.11×10^{-2}
E2	26.35	1.78×10^{-2}
F2	31.47	2.10×10^{-2}

Table S7. The glass transition temperatures (T_g) and crosslinking density of EWOA/EWOMA-TEGDMA resins with different dosages of TEGDMA.

Sample	Glass transition temperature (T_g, °C)	Crosslink density (mol/cm^3)
A1	24.92	2.23×10^{-3}
A2	21.65	2.41×10^{-3}
A3	19.51	1.94×10^{-2}
MA1	24.51	1.13×10^{-2}
MA2	20.95	1.19×10^{-2}
MA3	19.77	3.87×10^{-2}

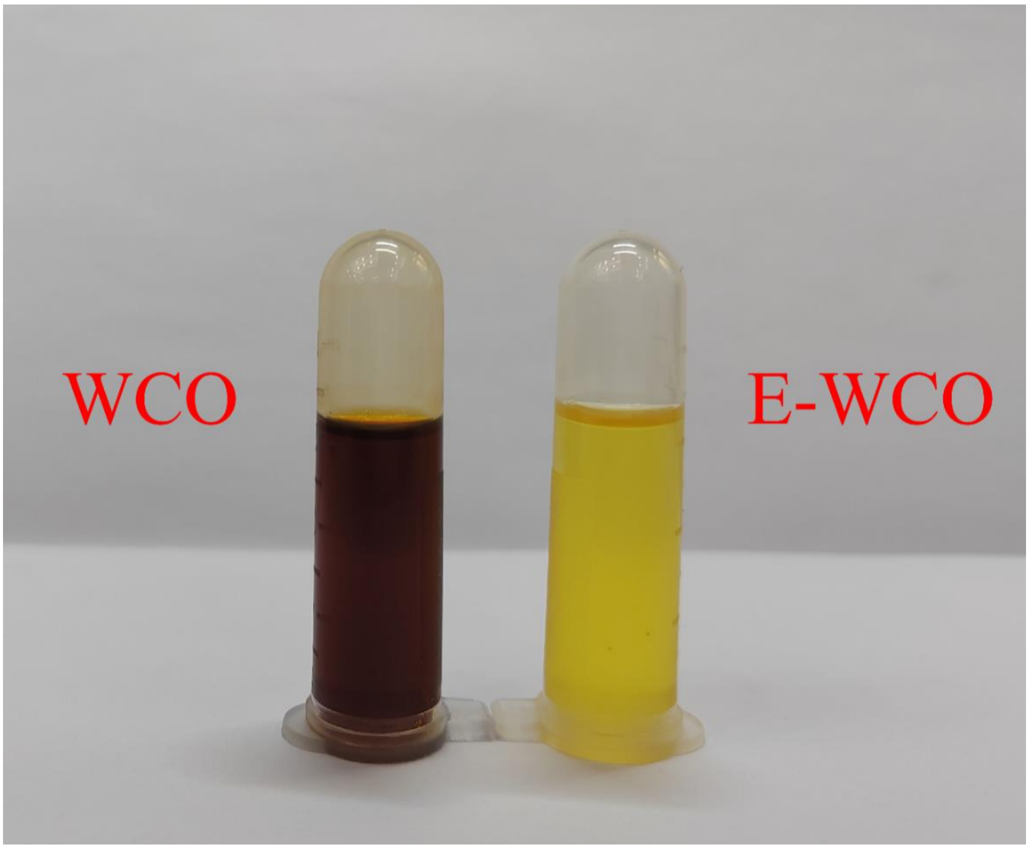

Figure S1. Photograph of WCO and E-WCO showing the difference in their colors.

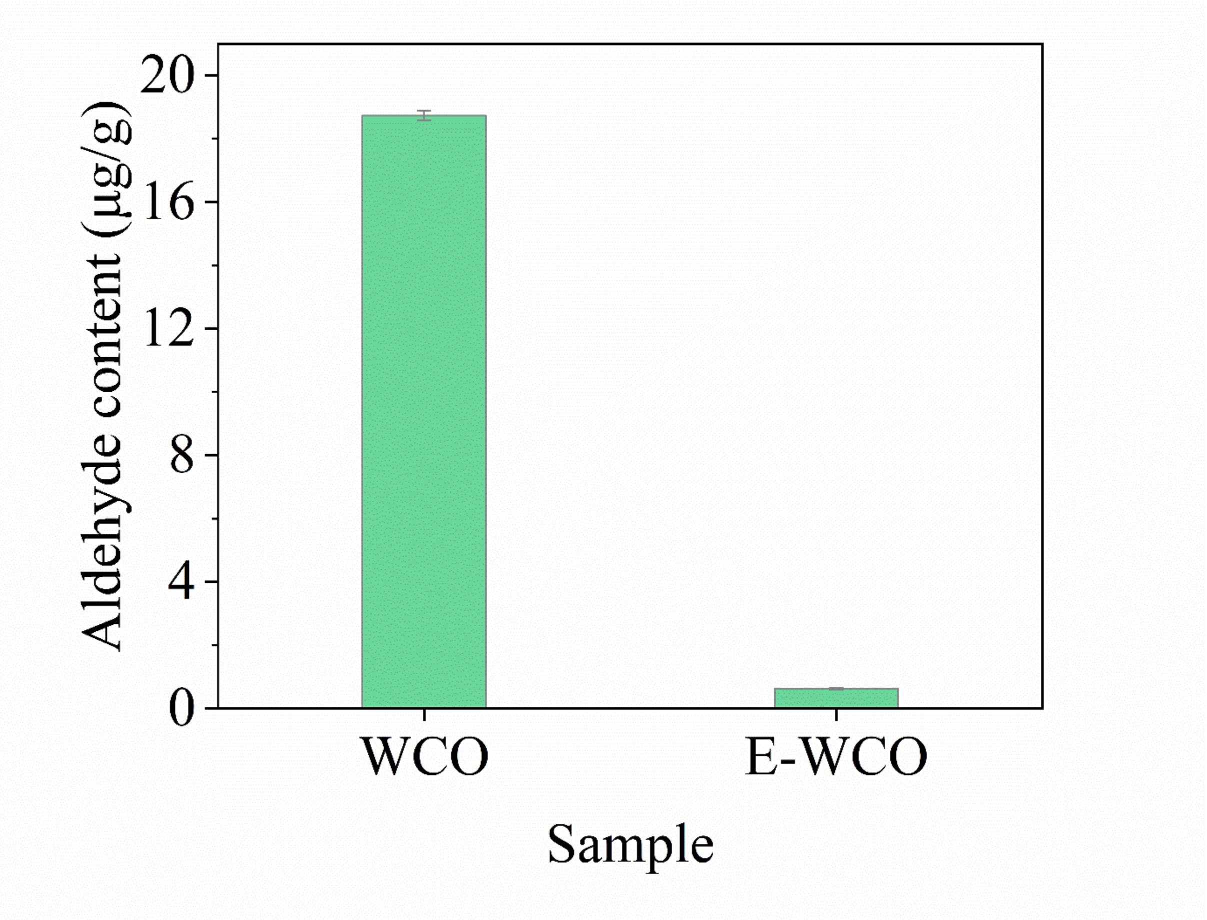

Figure S2. The aldehyde contents of WCO and E-WCO.

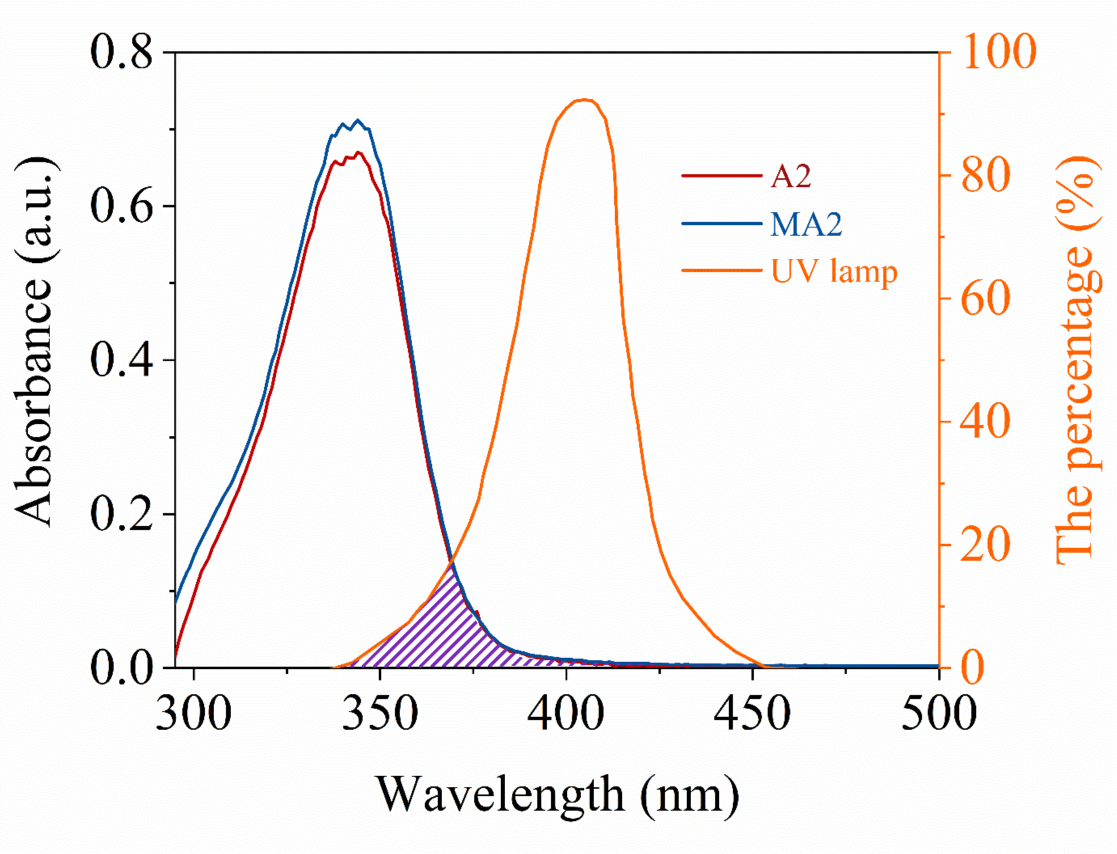

Figure S3. The absorption spectrum of the WCO-based photocurable resin (A2: blue, MA2: red) and the emission spectrum of the LCD curing light sources (orange).

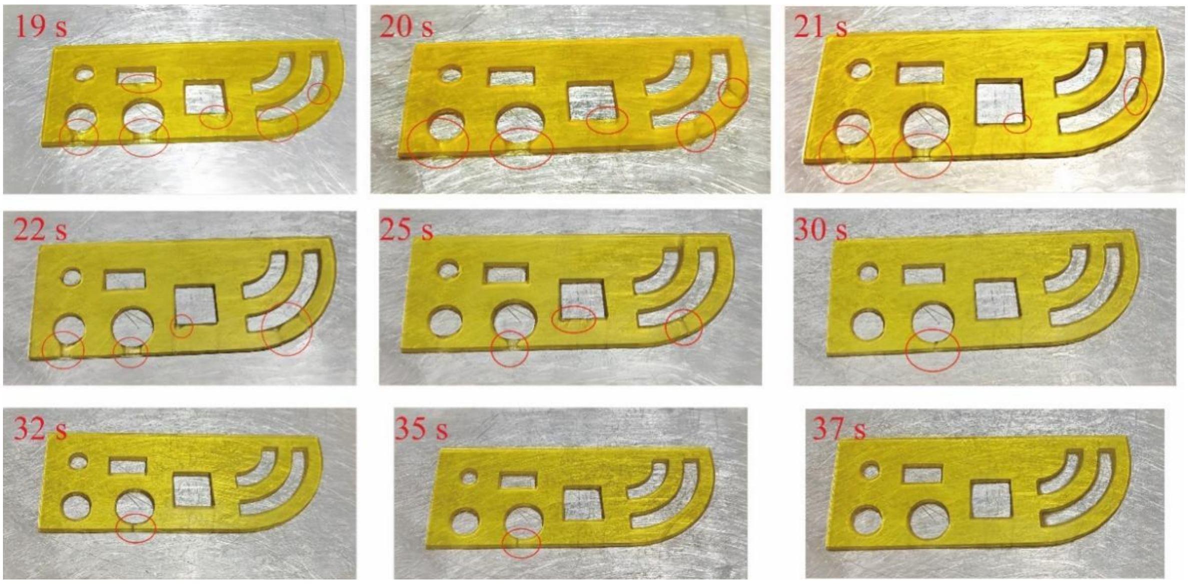

Figure S4. The influence of exposure time on the 4D-printed product.

The optimization of the normal exposure time followed the principle that the quality of the 4D-printed product was prior, while the printing efficiency should also be given consideration. As shown in Figure S3, the WCO-based photocurable resin could not achieve complete curing and would cause print defects when the exposure time was less than 37 s. Thus the optimized exposure time was set as 37 s for each layer in consideration of printing efficiency.

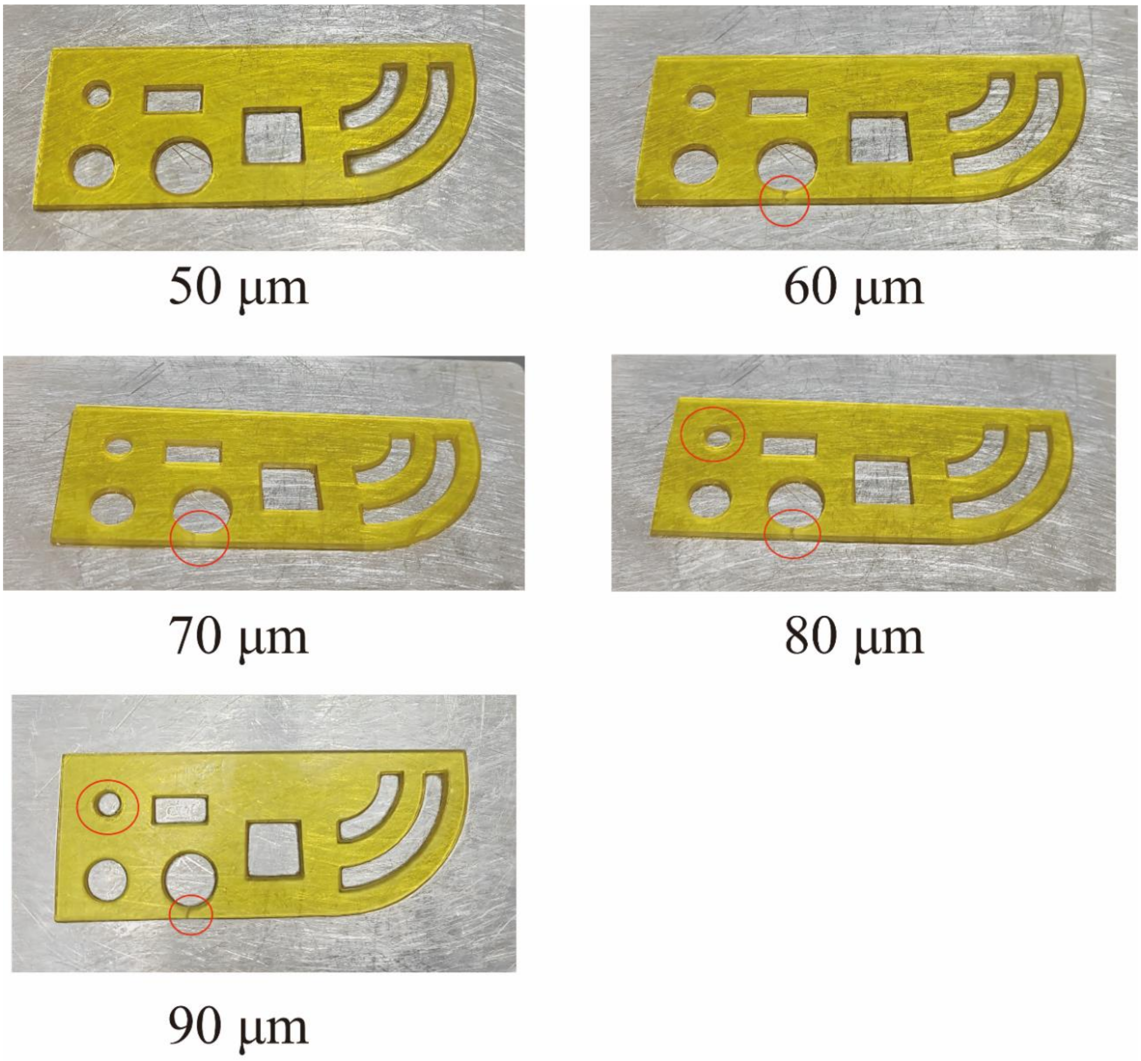

Figure S5. The influence of layer thickness on the 4D-printed product.

The optimization of the layer thickness followed the principle that the quality of the 4D-printed product was prior, while the printing efficiency should also be given consideration. As shown in Figure S4, the WCO-based photocurable resin could not achieve complete curing and would cause print defects when the layer thickness was more than 50 μm. Thus the optimized exposure time was set as 50 μm for each layer in consideration of printing efficiency.

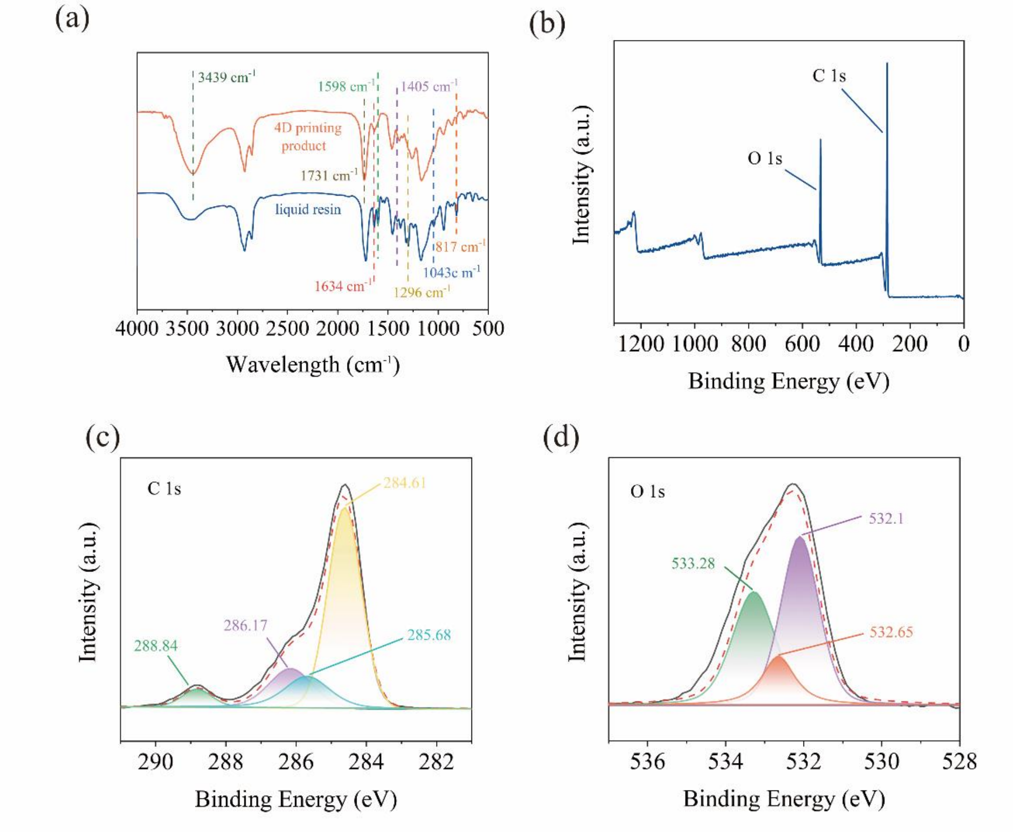

Figure S6. (a) IR spectra of 4D-printed product and liquid resin of MA2; (b-d) The full XPS (b), C1s (c), and O1s (d) high-resolution XPS spectra of 4D-printed product of MA2 resin.

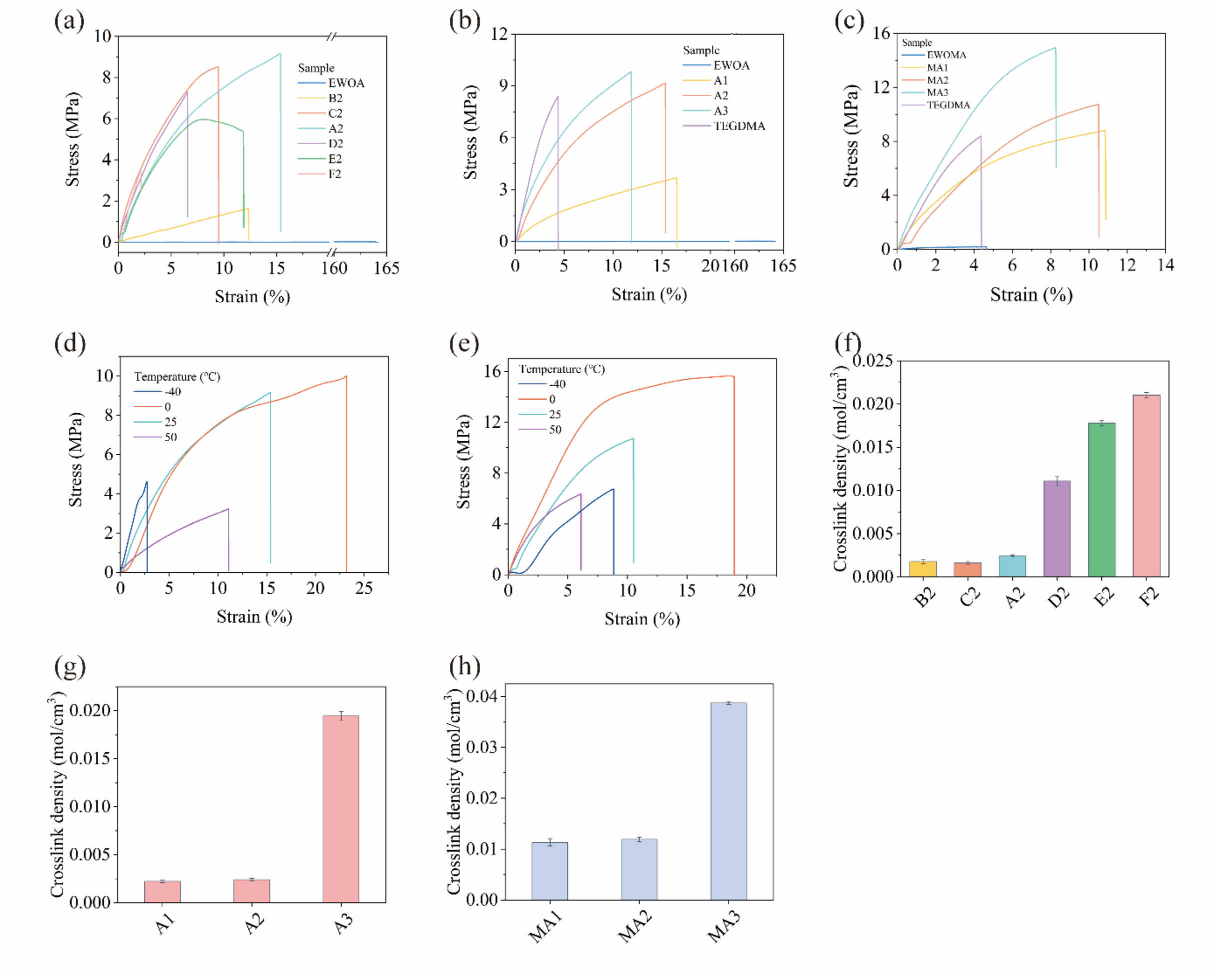

Figure S7. (a) The stress-strain curves of pure EWOA and WCO-based 4D-printable resins composed of EWOA and different diacrylate molecules; (b) The stress-strain curves of pure EWOA, pure TEGDMA and EWOA-TEGDMA resin with different dosages of TEGDMA; (c) The stress-strain curves of of pure EWOMA, pure TEGDMA and EWOMA-TEGDMA resins with different dosages of TEGDMA; (d-e) The stress-strain curves of A2 (d) and MA (e) resins at different temperatures; (f) The crosslinking density of WCO-based 4D-printable resins composed of EWOA and different diacrylate molecules; (g) The crosslinking density of EWOA-TEGDMA resins with different dosages of TEGDMA; (h) The crosslinking density of EWOMA-TEGDMA resins with different dosages of TEGDMA.

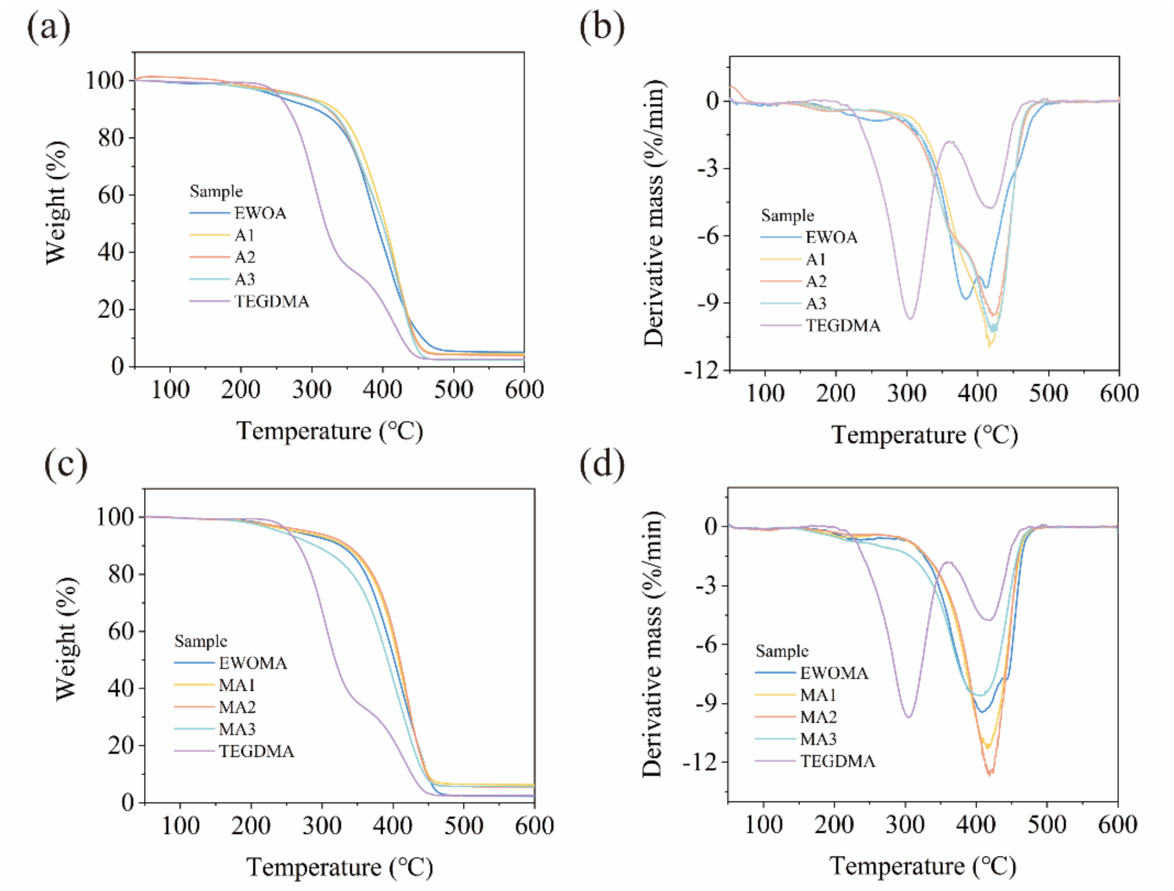

Figure S8. (a, b) The TG and DTG curves of pure EWOA, pure TEGDMA and EWOA-TEGDMA resins with different dosages of TEGDMA; (c, d) The TG and DTG curves of pure EWOMA, pure TEGDMA and EWOMA-TEGDMA resins with different dosages of TEGDMA.

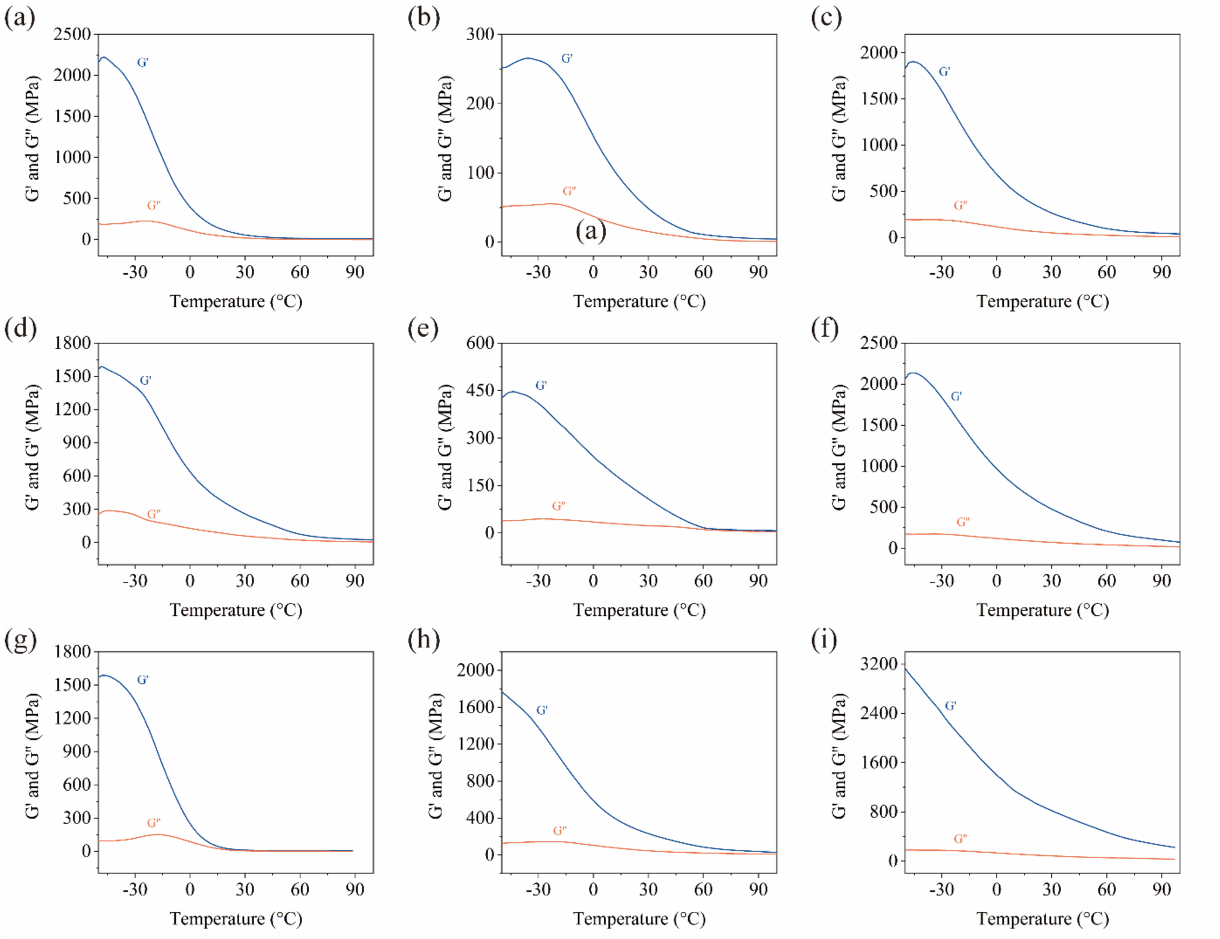

Figure S9. (a-f) The DMA spectra of EWOA-TEGDMA and EWOMA-TEGDMA resins with different dosages of TEGDMA: (a) A1, (b) A2, (c) A3, (d) MA1, (e) MA2, (f) MA3; (g-i) The DMA spectra of pure EWOA (g), EWOMA (h) and TEGDMA (i).

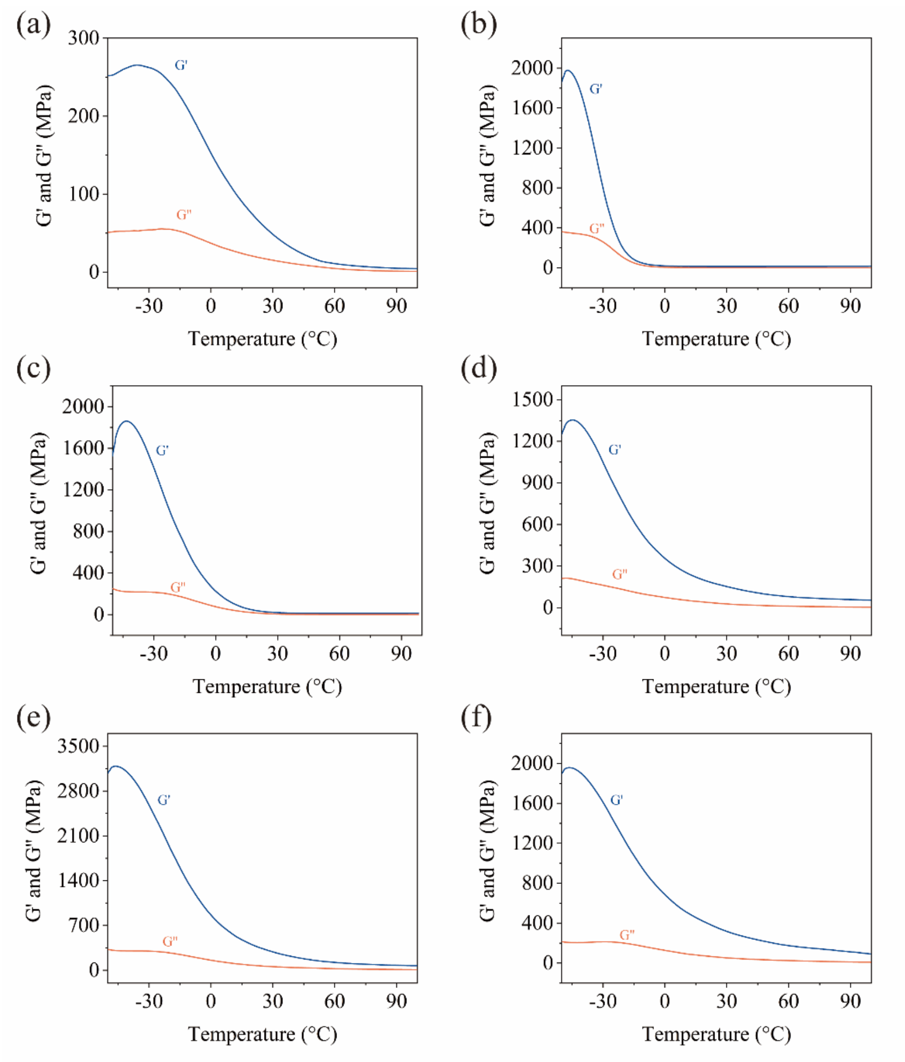

Figure S10. The dynamic mechanical temperature spectra of EWOA-R resin with different second monomers (both the dosages of EWOA were 100 g in this series)): (a) A2, (b) B2, (c) C2, (d) D2, (e) E2, (f) F2.

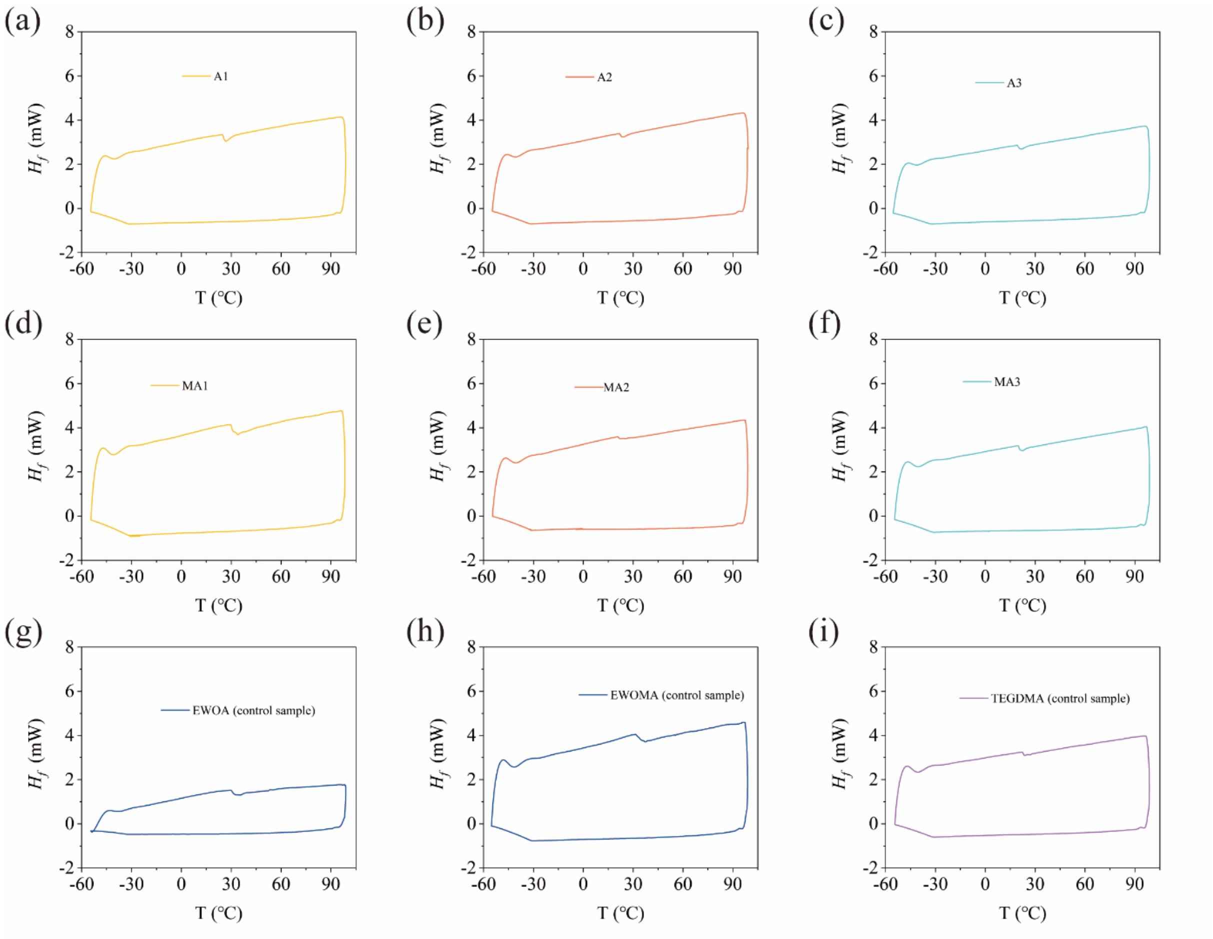

Figure S11. (a-f) The DSC spectra of EWOA-TEGDMA and EWOMA-TEGDMA resin with different dosages of TEGDMA: (a) A1, (b) A2, (c) A3, (d) MA1, (e) MA2, (f) MA3; (g-i) The DSC spectra of pure EWOA (g), EWOMA (h) and TEGDMA (i).

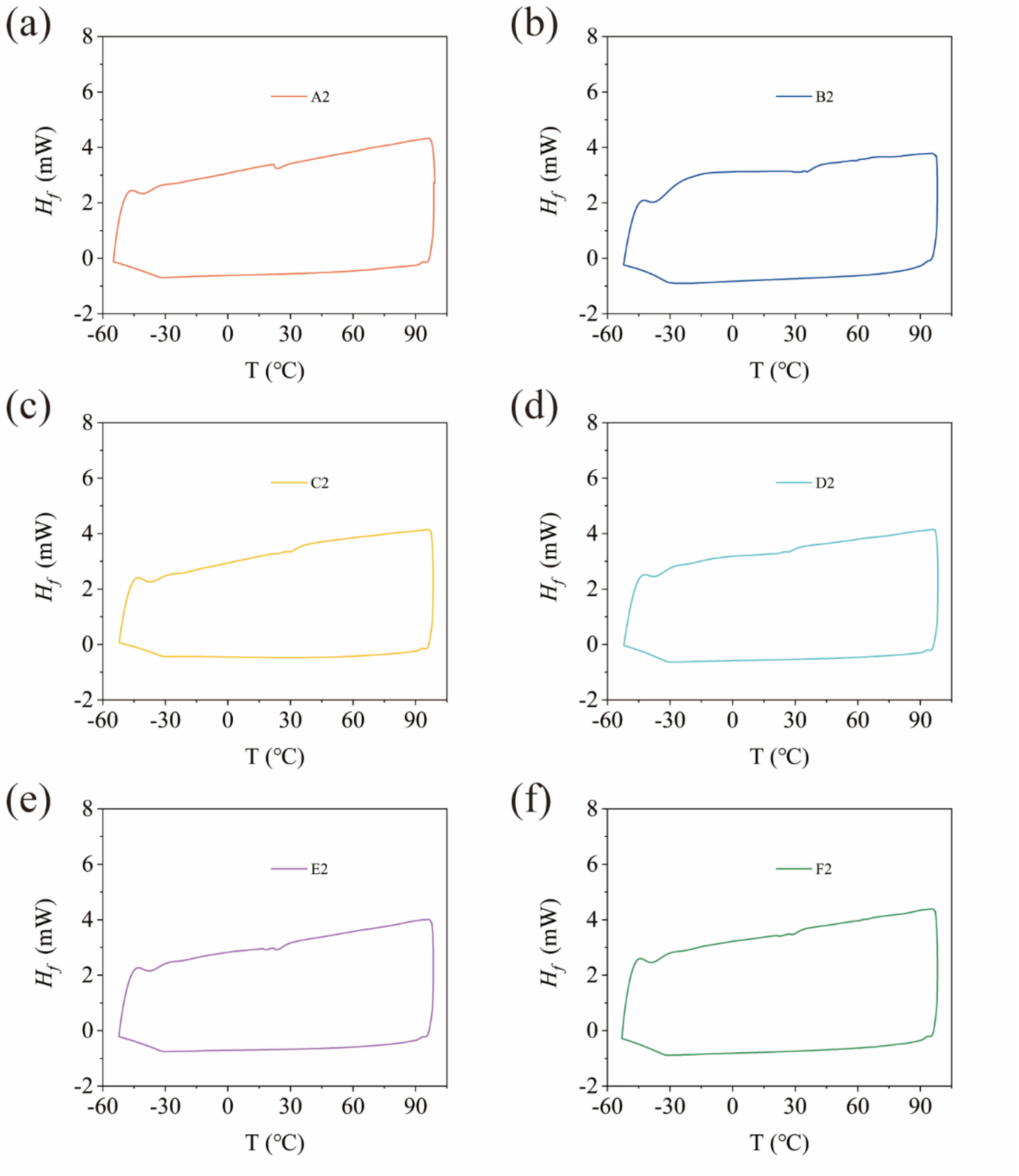

Figure S12. The DSC spectra of WCO-based 4D-printable resins composed of EWOA and different diacrylate molecules: (a) A2, (b)B2, (c) C2, (d) D2, (e) E2, (f) F2.

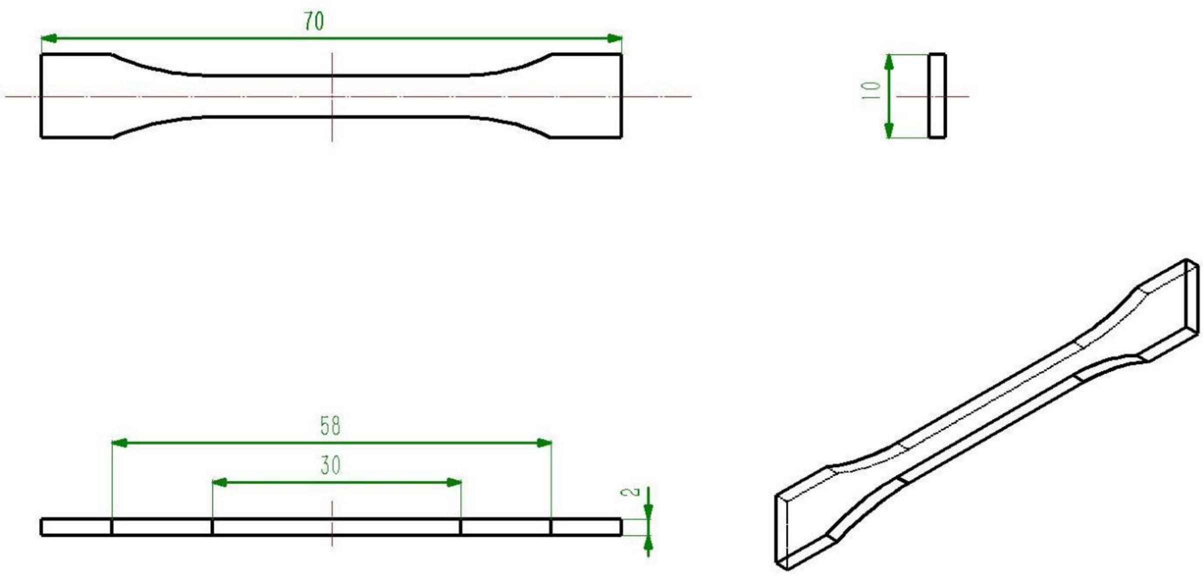

Figure S13. CAD model diagram of 1BA dumbbell-shaped spline according to the China Standard GB/T 1040.2-2006.
